# Supplementary material for: Observation of an electronic order along [110] direction in FeSe
Source: Nat Commun. 2021 Mar 2;12:1385. doi: 10.1038/s41467-021-21318-w (PMC7925548; doi:10.1038/s41467-021-21318-w)
Supplement: Supplementary file 1 — Supplementary Information [file 41467_2021_21318_MOESM1_ESM.pdf]

# Supplemental Materials for:

## Observation of an electronic order along [110] direction in FeSe

Kunliang Bu,[1] Wenhao Zhang,[1] Ying Fei,[1] Yuan Zheng,[1] Fangzhou Ai,[1]
Zongxiu Wu,[1] Qisi Wang,[2] Hongliang Wo,[2] Jun Zhao,[2,3] and Yi Yin[1,3,*]

[1]*Zhejiang Province Key Laboratory of Quantum Technology and Device, Department of Physics, Zhejiang University, Hangzhou 310027, China*

[2]*State Key Laboratory of Surface Physics and Department of Physics, Fudan Univresity, Shanghai 200433, China*

[3]*Collaborative Innovation Center of Advanced Microstructures, Nanjing University, Nanjing 210093, China*

[*] yiyin@zju.edu.cn

## Supplementary Note 1: Quality of the single crystal sample of bulk FeSe

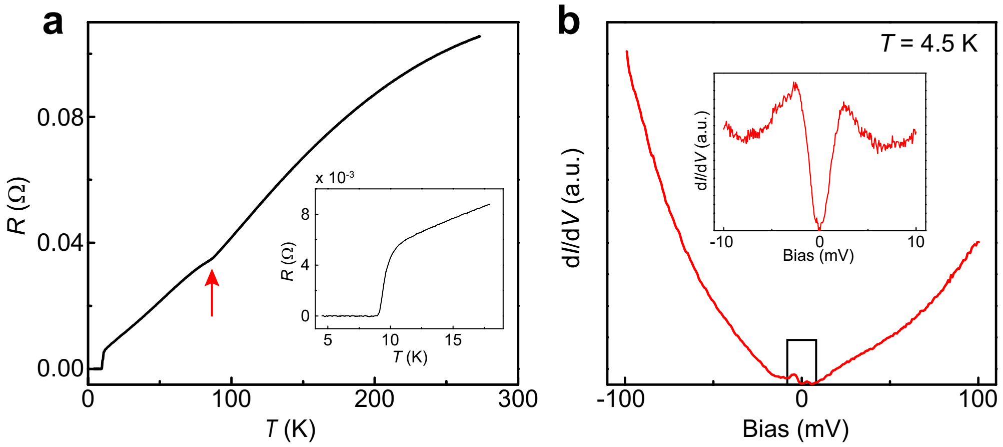


**Supplementary Figure** 1. **Resistance curve and d$I$/d$V$ spectrum of FeSe. a** Resistance curve of the FeSe sample measured upon warming. The red arrow indicates the critical temperature of the tetragonal to orthorhombic structural transition. Inset shows data around the superconducting critical temperature $T_c$. **b** d$I$/d$V$ spectrum of FeSe with a large energy range taken at 4.5 K. The tunneling condition is $V_b$ = 20 mV and $I_s$ = 100 pA. Inset shows the spectrum around the Fermi level, with an obvious supercondicting gap.

Supplementary Figure 1a shows the temperature-dependent resistance curve for the single crystal sample of bulk FeSe. A kink feature at around 90 K corresponds to the tetragonal to orthorhombic structural transition. The resistance quickly drops to a zero value at $T_c$ = 8.96 K, below which the sample enters the superconducting phase. As shown in the inset, the sharp resistance drop is measured with a transition width of 1.7 K. The residual resistance ratio ($R_{273\mathrm{K}}/R_{11\mathrm{K}}$) is ∼20, indicating a high quality of the sample. Supplementary Figure 1b shows a typical d$I$/d$V$ spectrum of FeSe with a large energy range at 4.5 K. As shown in the inset, a superconducting gap of $\Delta$ = 2.5 meV can be determined. Both the critical temperature and superconducting gap are consistent with previous experimental results [1, 2].

## Supplementary Note 2: Tip condition optimized for experiments

Here we discuss how to optimize the tip condition for the specified STM experiment. The conductivity of FeSe is relatively weak compared with other materials, therefore the oscillation of atomic lattice is rather weak in the topographic image. Both a close tip-sample distance and a sharp tip are beneficial for us to obtain a clear topographic image.

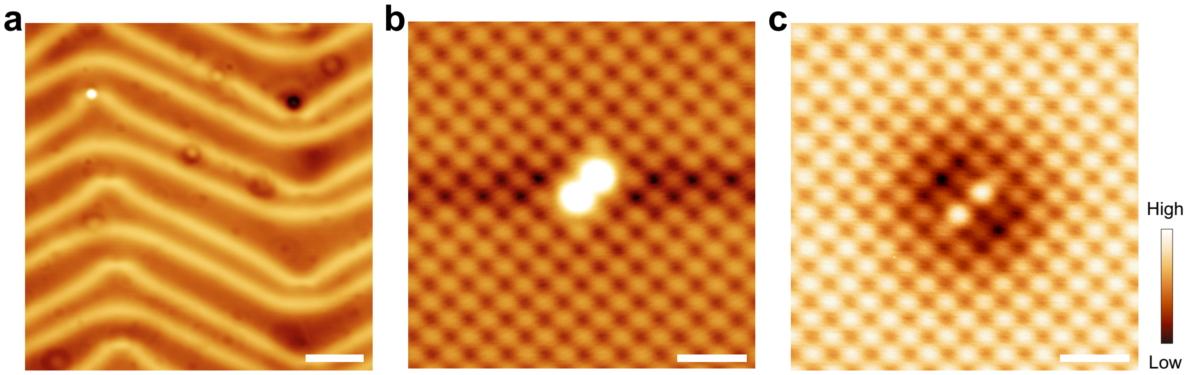


**Supplementary Figure 2. Tip optimization for FeSe measurement. a** A 30 nm × 30 nm STM image of a Au (111) surface (scale bar: 5 nm). The tunneling condition is $V_b$ = -600 mV and $I_s$ = 200 pA. **b** A 5 nm × 5 nm STM image of a dumbbell defect under $V_b$ = 100 mV and $I_s$ = 20 pA (scale bar: 1 nm). **c** A 5 nm × 5 nm STM image of the same dumbbell defect under $V_b$ = -100 mV and $I_s$ = 20 pA (scale bar: 1 nm).

On the other hand, the measurement requires a wide energy range (or bias voltage range). A large tip-sample distance is beneficial for this purpose, to avoid a possible signal overload. With the two contradictory requirements, we need a sharp tip and maintain a balanced tip-sample distance in our STM experiment.

To detect the ordered states of FeSe, we need to avoid any anisotropy induced by the tip [3]. Supplementary Figure 2 shows some scanning topographies for a typical tip with good condition. In our experiment, a tungsten tip is carefully treated on a Au (111) surface. In Supplementary Figure 2a, the small round adatom of Au on the left-up corner indicates a sharp isotropic tip after treatment. The dumbbell defect in FeSe is also a touchstone for the tip condition. In the topography, the apparent height of a dumbbell defect is usually much higher than the oscillation of Se lattice. Any feature on the tip apex can be conspicuous on the dumbbell defect in the topography. Supplementary Figures 2b and 2c show the topographies around a dumbbell defect under a positive and negative bias voltage, respectively. Here the junction resistance is also much larger than that in previous STM reports about FeSe [2, 4]. An isotropic tip is represented by the round spot of the dumbbell defect under both bias-voltage polarities. A sharp tip is represented by the clear dumbbell feature under such a large tip-sample distance. Before each measurement, we carefully treat the tip until getting a sharp and isotropic tip. To ensure an isotropic tip in the whole measurement

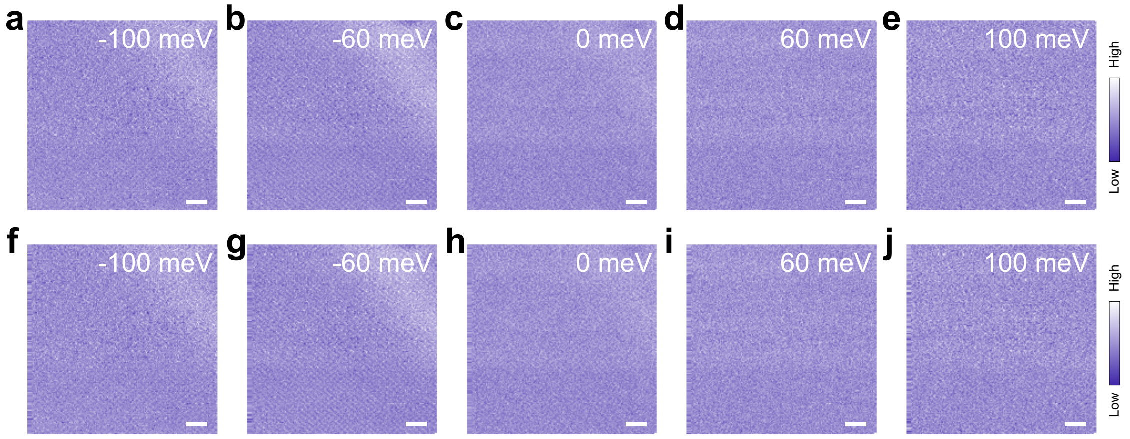


**Supplementary Figure 3. Conductance maps before and after the drift correction process. a-e** Unprocessed conductance maps. **f-j** Conductance maps after applying the Lawler-Fujita algorithm. Scale bars are 2 nm.

process, we also measure and check a topography including dumbbell defects after finishing the grid spectroscopy.

## Supplementary Note 3: Comparison with the random groups of Fe atoms

As mentioned in the main text, we apply a Lawler-Fujita algorithm to remove the thermal drift in the raw data. The conductance maps at different energies are drift corrected with the topography simultaneously. Supplementary Figure 3 shows the conductance maps before and after applying the Lawler-Fujita algorithm. Since a pure area is selected for the measurement, little information can be obtained from the conductance maps with the naked eyes. The sites of each atom can be precisely located after the drift correction of the topography. In Supplementary Figure 4a, the sites of $Fe_1$ and $Fe_2$ atoms are superimposed on the topography, a partial image of Fig. 2a in the main text. Each $\mathrm{d}I/\mathrm{d}V$ spectrum of Fe atom can be extracted from the grid spectroscopy taken simultaneously with the topography. We extract and average all the $\mathrm{d}I/\mathrm{d}V$ spectra taken at sites of $Fe_1$ and $Fe_2$ atoms, respectively. The averaged spectra for $Fe_1$ and $Fe_2$ are shown in Supplementary Figure 4b. As shown in the inset, a small but non-zero difference can be discerned between the averaged spectra of $Fe_1$ and $Fe_2$. We also classify all Fe atoms into two groups randomly, whose sites are labeled and superimposed in Supplementary Figure 4c. In the averaged spectra, no difference can be discerned as shown in the inset of Supplementary Figure 4d. In an area of 18 nm $\times$ 18 nm, there are over 4500 Fe atoms in the field of view (FOV). The averaged spectrum is from

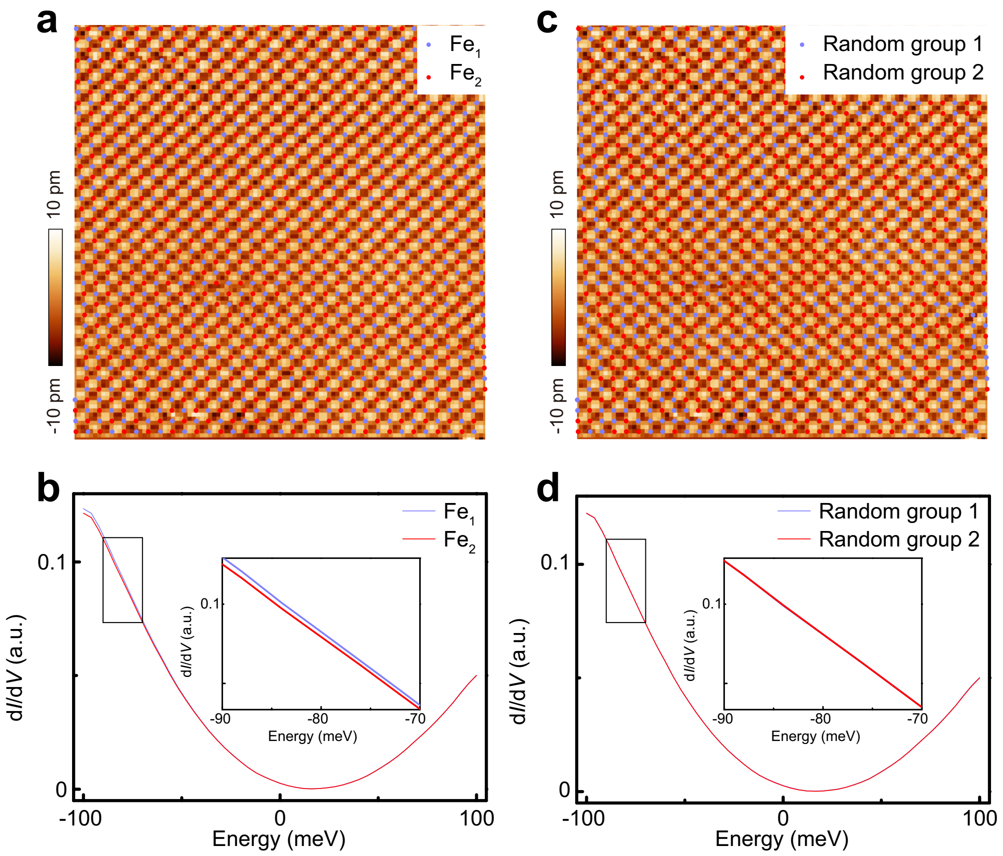


**Supplementary Figure** 4. **Classification of Fe atoms. a** A partial topography of Fig. 2a in the main text, with sites of $Fe_1$ and $Fe_2$ atoms superimposed on it. **b** Averaged $\mathrm{d}I/\mathrm{d}V$ spectra of $Fe_1$ and $Fe_2$ atoms. Inset shows the enlargement of the black box. **c** A topography at the same area as **a**. The Fe atoms are now randomly classified into two groups, as shown by the superimposed sites. **d** Averaged $\mathrm{d}I/\mathrm{d}V$ spectra of two groups of random Fe atoms. Inset shows the enlargement of the black box.

more than 2250 raw spectra. Despite the small difference between the averaged spectra of $Fe_1$ and $Fe_2$, it is a robust physical signal instead of noisy fluctuations.

**Supplementary Note 4: Filtering procedure to obtain the local [110] electronic order**

The local [110] electronic order within each unit cell is represented by the difference between the differential conductance at sites of $Fe_1$ and $Fe_2$ atoms. An example of this result is shown in Supplementary Figure 5. Due to the unavoidable background noise, there are perturbations in the local order maps (Supplementary Figures 5a and 5b). The intra-unit cell information is mainly from the signal at wave vectors near $\mathbf{Q}_x$ and $\mathbf{Q}_y$ in Fourier-transformed

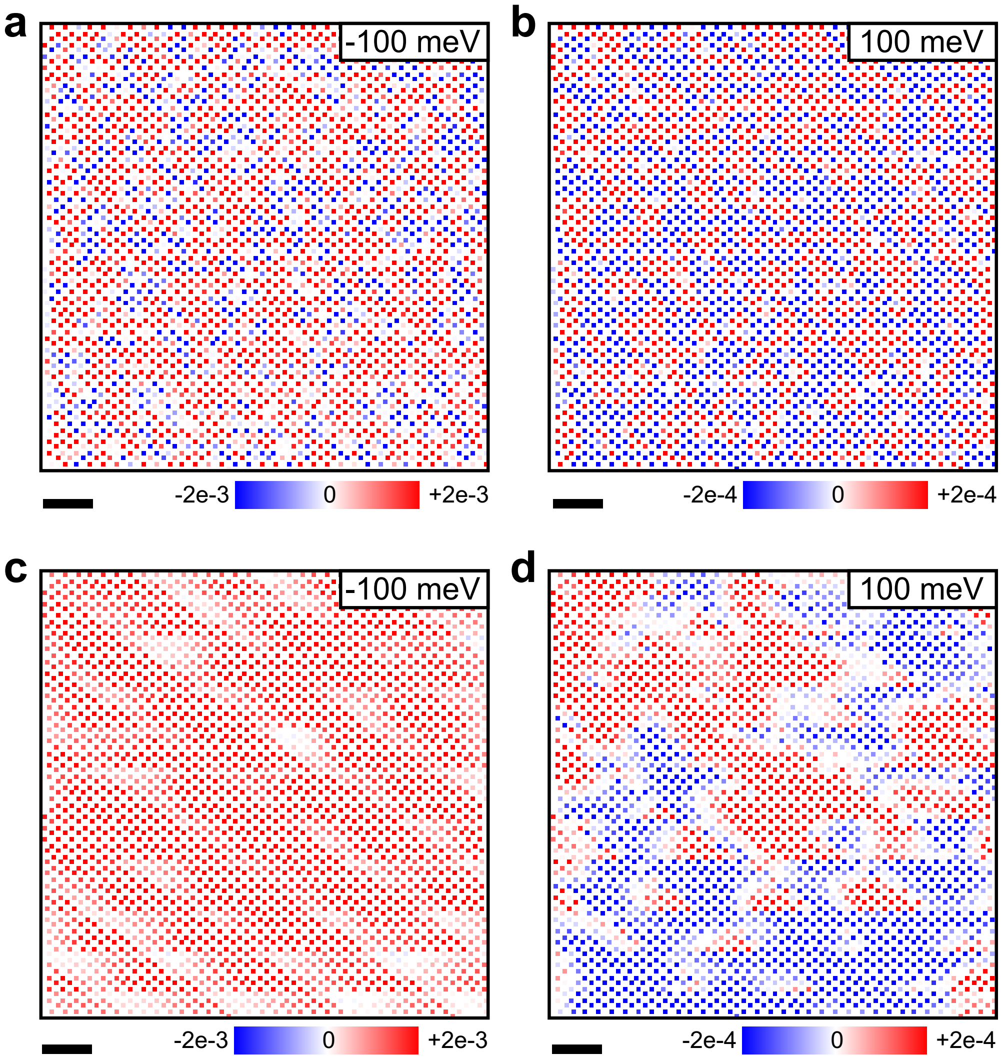


**Supplementary Figure** 5. **Comparison of the local order before and after the Gaussian filtration.** The local [110] electronic order before (**a** and **b**) and after (**c** and **d**) the Gaussian filtration at two representative energies. Scale bars are 2 nm.

maps, while the long range background noise have wave vectors far away from $\mathbf{Q}_x$ and $\mathbf{Q}_y$. We apply a Gaussian mask around each Bragg peaks of $\mathbf{Q}_x/\mathbf{Q}_y$ to filter out background noise in differential conductance maps [5]. In Supplementary Figures 5c and 5d, we show the local order calculated from the filtered differential conductance maps. The background noise is reasonably removed after the filtration. Here a slight filter has been applied with a cutoff size of $\Lambda^{-1} = 1$ nm. The criterion for the critical filter size is that the obtained local order patterns would not change with further increase of the filter radius. The filtered local order map can be understood as that each single point value is averaged over an area of the

filter size. In this filtered local order map, domains larger than 1 nm will not be affected by the filtration while features smaller than 1 nm is physically insignificant (like a tiny island smaller than 1 nm). We apply the same filtering parameter for data analysis at both liquid nitrogen and liquid helium temperatures.

**Supplementary Note 5: Robust [110] electronic order against the setup condition**

The charge order along the [110] direction in FeSe can be reproduced by different tips, on different samples, and under different tunneling conditions. Supplementary Figure 6a is a topography obtained from the same sample and by the same tip as Fig. 2a in the main text. In Supplementary Figure 6d, the difference between the averaged d$I$/d$V$ spectra of $Fe_1$ and $Fe_2$ shows a trend similar to that in Fig. 2c, although the pixel density is different in two measurements. The similar trend of the collective [110] order also indicates that the pixel density is large enough for the extraction of the [110] electronic order.

Supplementary Figures 6f-o are two datasets taken with two different samples and by two different tips. The tunneling conditions are set at $V_b$ = 20 mV, $I_s$ = 10 pA, and $V_b$ = -20 mV, $I_s$ = 20 pA, respectively. From the nonlinear $I - V$ curves (insets in Supplementary Figures 6f and 6k), these two setup conditions roughly correspond to the tunneling with $V_b$ = 100 mV and $I_s$ = 120 pA. We could infer that the tip-sample distance in two datasets are similar to each other, but shorter than that in Supplementary Figures 6a-e. With a shorter tip-sample distance and a positive bias voltage, the magnitude of the [110] electronic order in Supplementary Figure 6i is almost two times larger than that in Supplementary Figure 6d. In contrast, due to the normalization of the density of states from Fermi energy to $eV_b$, the non-zero [110] electronic order at -20 mV in Supplementary Figure 6i is suppressed in Supplementary Figure 6n, by adjusting the relative tip height on $Fe_1$ and $Fe_2$ sites. Although the whole [110] electronic order is suppressed in Supplementary Figure 6n, the trend of the order is still similar to that in Supplementary Figures 6d and 6i. We cannot define an absolute normalization condition in the d$I$/d$V$ measurement. One reasonable approach is to set the bias voltage at which the order is close to zero. For measurement at 77 K, $V_b$ = 100 mV and $I_s$ = 100 pA is a good choice of setup condition.

Because the tip conductance is various for different tips, the tip-sample distance may be different even when the setup condition is the same. Supplementary Figures 6p-y are two datasets taken on the same area of a sample, by a special tip, and under two different setup

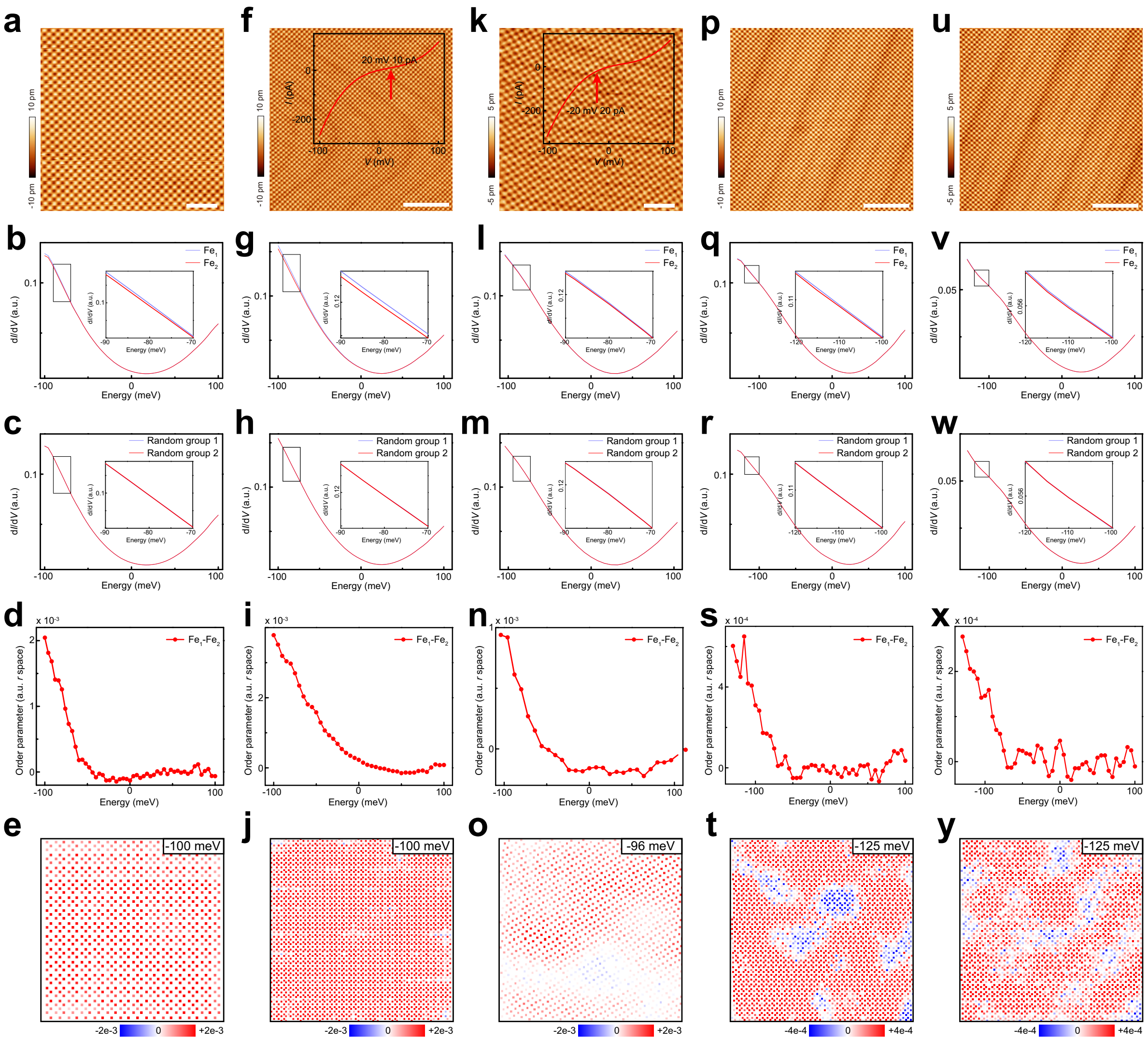

**Supplementary Figure 6. [110] electronic order measured under different setup conditions. a** A 12 nm × 12 nm topography taken under $V_b$ = 100 mV and $I_s$ = 100 pA, with 160 × 160 pixels (scale bar: 2 nm). The results in **b-e** are from the measurement taken in this filed of view (FOV). **b, c** Averaged spectra for $Fe_1$ and $Fe_2$ (**b**) and two groups of random Fe atoms (**c**). **d** The difference between the averaged spectra of $Fe_1$ and $Fe_2$. **e** Local order map at -100 meV. **f-j** Similar as **a-e**, but with analysis in the FOV in **f** (20 nm × 20 nm, $V_b$ = 20 mV, $I_s$ = 10 pA, 200×200 pixels, scale bar: 5 nm). **k-o** Similar as **a-e**, but with analysis in the FOV in **k** (12 nm × 12 nm, $V_b$ = -20 mV, $I_s$ = 20 pA, 120×120 pixels, scale bar: 2 nm). Insets in **f** and **k** show the corresponding $I-V$ curves under the tunneling conditions indicated by the red arrows, respectively. **p-t** Similar as **a-e**, but with analysis in the FOV in **p** (20 nm × 20 nm, $V_b$ = 100 mV, $I_s$ = 100 pA, 200×200 pixels, scale bar: 5 nm). **u-y** Similar as **a-e**, but with analysis in the FOV in **u** (20 nm × 20 nm, $V_b$ = -100 mV, $I_s$ = 100 pA, 200×200 pixels, scale bar: 5 nm). All these datasets were taken at 77 K.

conditions ($V_b$ = 100 mV, $I_s$ = 100 pA, and $V_b$ = -100 mV, $I_s$ = 100 pA). In Supplementary Figure 6s, the [110] electronic order is relatively weak compared with that in Supplementary Figure 6d, despite under the same setup condition. We speculate that the weaker [110] electronic order is due to the larger tip-sample distance, which is possibly induced by the different tip conductance. The setup condition of $V_b$ = -100 mV and $I_s$ = 100 pA roughly corresponds to a tunneling with $V_b$ = 100 mV and $I_s$ = 50 pA. The larger tip-sample distance mainly leads to the slightly suppressed [110] electronic order in Supplementary Figure 6x compared with that in Supplementary Figure 6s. The weak [110] electronic order at -100 mV makes the normalization a small effect.

Among all these datasets, we show the averaged $dI/dV$ spectra for $Fe_1$ and $Fe_2$ as well as two groups of random Fe atoms (Supplementary Figures 6b-c, 6g-h, 6l-m, 6q-r, and 6v-w). The order strength is also consistent with the corresponding local order map. Weak noise patterns are visible in Supplementary Figures 6f, 6k, 6p and 6u, due to the small oscillation of the Se atoms. However, these long range stripes (with wavelength of several nanometers) have little effect on the obtained [110] order, because there are no overlaps of wave vectors between noise peaks and Bragg peaks (Supplementary Note 4). The consistency of the order trend under different conditions indicates a robust [110] electronic order at 77 K.

**Supplementary Note 6: Evolution of the local [110] electronic order at 4.5 K**

Supplementary Figure 7 shows the energy dependence of the local [110] electronic order for the data in Fig. 4a in the main text. At low energies, the positive order (red dots) and negative order (blue dots) are roughly separated by a boundary along the diagonal direction (Supplementary Figures 7a-c). With the increase of energy, the strength of the local order first decreases, becoming one order of magnitude smaller when the energy approaches [-40, 40] meV. At the same time, the boundary of the positive and negative orders becomes blurred. The red and blue patches interpenetrate each other to form nanometer-sized domains. With further increase of the energy, the value of local order gradually increases (Supplementary Figures 7j-l). The boundary of the positive and negative orders becomes clear again. For $|E| > 80$ meV, the positive and negative orders are similarly separated by a boundary along the diagonal direction. Thus we divide the field of view into two domains to calculate the collective order, respectively.

**Supplementary Note 7: [110] electronic order reproduced at 4.5 K**

Supplementary Figure 8 is another example of the [110] electronic order at liquid helium

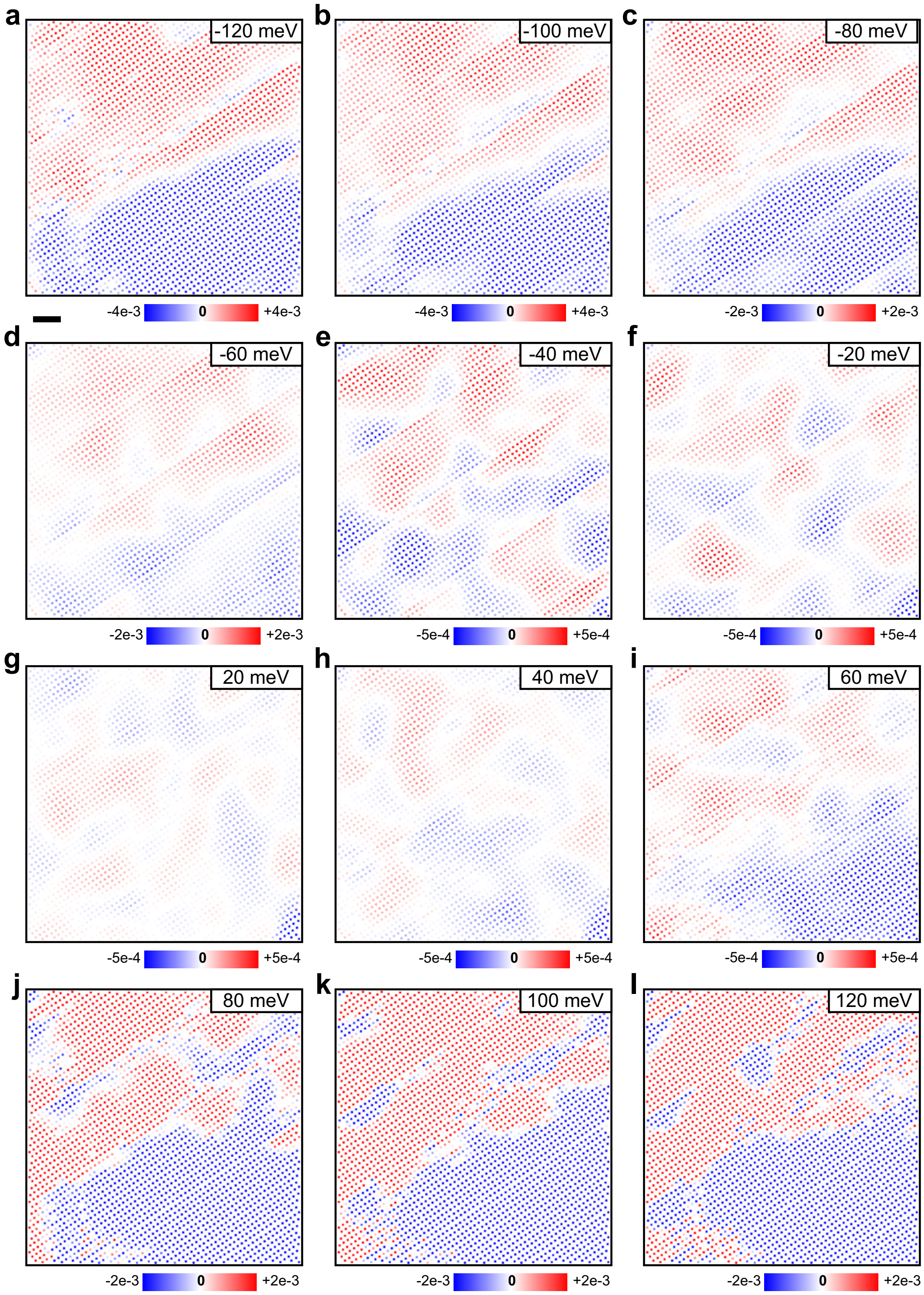


**Supplementary Figure 7. Evolution of the local [110] electronic order.** The energy dependent local [110] electronic order from -120 meV to 120 meV. The local order maps are obtained from the Gaussian-filtered differential conductance map with the filter size of $\Lambda^{-1} = 1$ nm. The color bars indicate the difference between the differential conductance at $Fe_1$ and $Fe_2$ sites within each intra-unit cell. The scale bars are 2 nm.

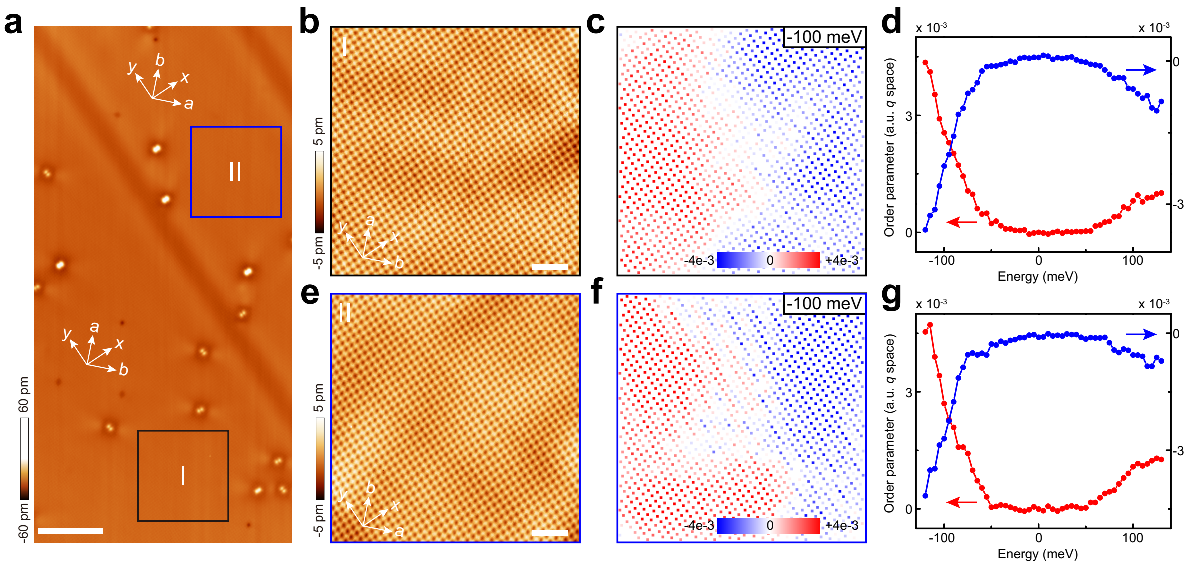


**Supplementary Figure** 8. **[110] electronic order near a twin boundary at 4.5 K. a** An 80 nm × 40 nm topography under $V_{\rm b}$ = 100 mV and $I_s$ = 20 pA (Scale bar: 10 nm). **b** The enlargement of the black square (14 nm × 14 nm, $V_{\rm b}$ = 20 mV, $I_s$ = 20 pA) in **a** (Scale bar: 2 nm). **c** Local [110] electronic order in the area of **b**, obtained from the Guassian filtered differential conductance map with a filter size of $\Lambda^{-1} = 1$ nm. **d** The collective [110] electronic order of the red and blue areas in **c**. **e-g** The same as that in **b-d** but with the area on another side of the twin boundary, as indicated by the blue square in **a**.

temperature. We select two clean areas on two sides of a twin boundary (TB). After the Gaussian filtration procedure applied to the differential conductance map, the local [110] electronic order is calculated and shown in Supplementary Figure 8c for the left side of the TB. Similar to Fig. 4a, the positive order and negative order are separated into two domains at -100 meV. Similar results can also be obtained at another side of the TB (Supplementary Figure 8f). We separately calculate the collective order of the domains with positive and negative orders, as shown in Supplementary Figures 8d and 8g. On one side of the TB, the collective order of different signs (corresponding to red and blue areas) follows the opposite trend. On both sides of the TB, the collective order of the same sign follows a similar trend. All these characteristics are consistent with the results in Fig. 4 in the main text.

In Supplementary Figure 9, we also show a measurement far from the twin boundary at 4.5 K. Under the setup condition $V_{\rm b}$ = 40 mV and $I$ = 20 pA, a grid spectroscopy is measured with a large energy range from -200 meV to 200 meV. The averaged spectra of

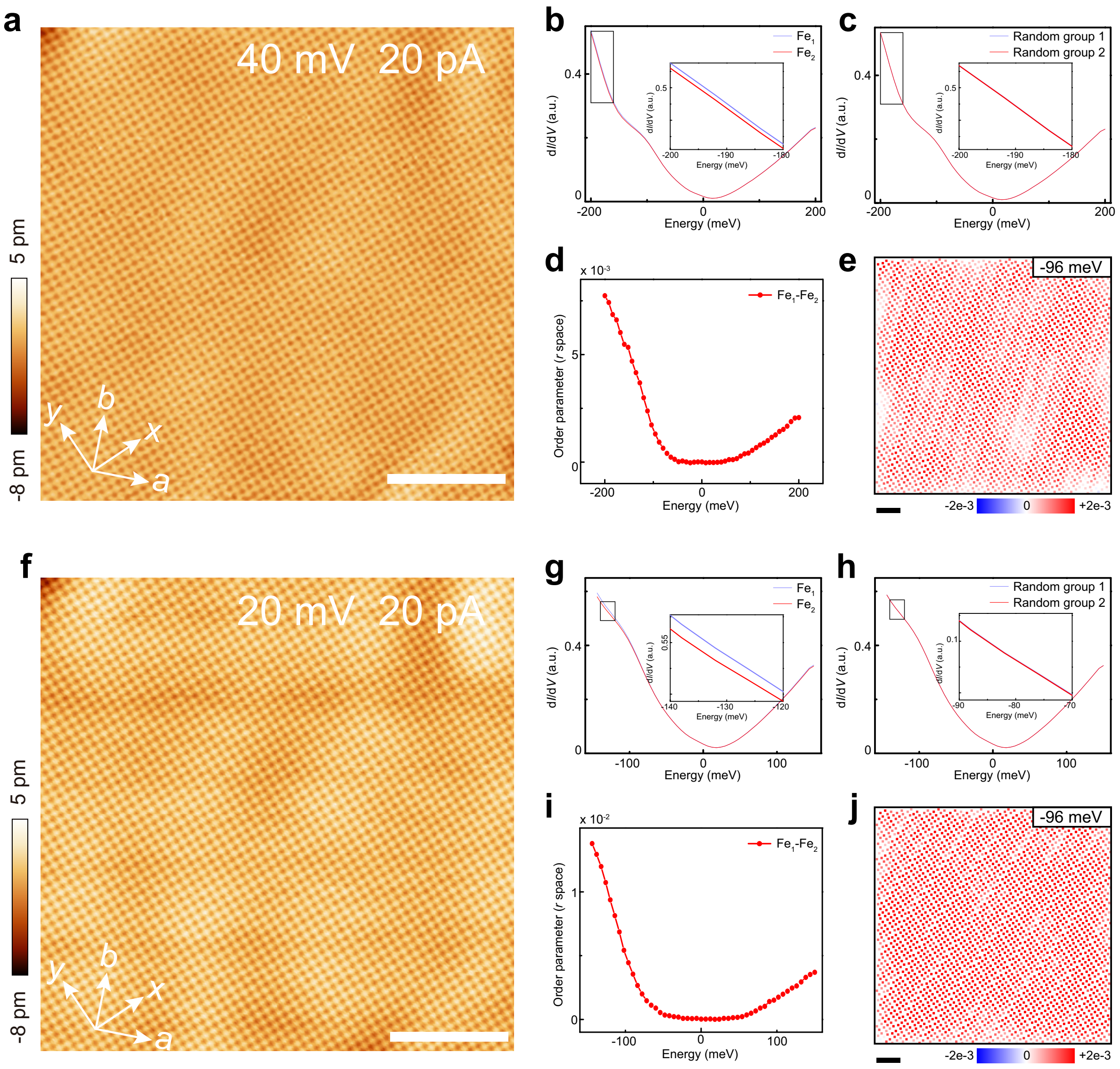


**Supplementary Figure** 9. **Robust [110] electronic order far from the twin boundary at 4.5 K. a** A 20 nm × 20 nm topography obtained with $V_b = 40$ mV and $I_s = 20$ pA (Scale bar: 5 nm). **b, c** The averaged spectra for $Fe_1$ and $Fe_2$ (**b**) and two groups of random Fe atoms (**c**). **d** The difference between the averaged spectra of $Fe_1$ and $Fe_2$ atoms. **e** The local order map at the energy of -96 meV. **f-j** The same area with **a** but with a different setup condition of $V_b = 20$ mV and $I_s = 20$ pA.

$Fe_1$ and $Fe_2$ atoms are shown in Supplementary Figure 9b, and the averaged spectra of two groups of random Fe atoms are shown in Supplementary Figure 9c. Due to the large energy interval (6 meV) in the grid spectroscopy measurement, in these spectra we cannot see the superconducting gap. A difference between the averaged spectra of $Fe_1$ and $Fe_2$ can be

discerned while no difference is found between the averaged spectra of two groups of random Fe atoms. Similar to the result in Fig. 4 and Supplementary Figure 8, a roughly [-40, 40] meV gap is reproduced for this [110] electronic order. Supplementary Figure 9e shows the local order map at -96 meV, retaining a robust [110] electronic order at 4.5 K. In the same field of view, we also measure a dense array (10 pixels per 1 nm) of grid spectroscopy under the setup condition $V_{\mathrm{b}} = 20$ mV and $I_s = 20$ pA. With a shorter tip-sample distance, the measured energy ranged is changed to [-150, 150] meV to avoid the overload signal. The difference between the averaged spectra of $Fe_1$ and $Fe_2$ can be clearly discerned. The [-40, 40] meV gap as well as the [110] robust local order are both well reproduced.

[1] Kasaharaa, S. *et al.* Field-induced superconducting phase of FeSe in the BCS-BEC cross-over. *Proc. Natl. Acad. Sci. USA* **111**, 16309-16313 (2014).

[2] Jiao, L. *et al.* Superconducting gap structure of FeSe, *Sci. Rep.* **7**, 44024 (2017).

[3] Lawler, M. J. *et al.* Intra-unit-cell electronic nematicity of the high-$T_c$ copper-oxide pseudogap states, *Nature* **466**, 347-351 (2010).

[4] Song, C.-L. *et al.* Suppression of superconductivity by twin boundaries in FeSe, *Phys. Rev. Lett.* **109**, 137004 (2012).

[5] Zheng, Y. *et al.* The study of electronic nematicity in an overdoped (Bi, $Pb)_2Sr_2CuO_{6+\delta}$ superconductor using scanning tunneling spectroscopy, *Sci. Rep.* **7**, 8059 (2017).
